# Enhanced multi-year predictability after El Niño and La Niña events

**DOI:** 10.1038/s41467-023-42113-9

**Published:** 2023-10-11

**Authors:** Yiling Liu, Markus. G. Donat, Matthew. H. England, Lisa. V. Alexander, Annette L. Hirsch, Carlos Delgado-Torres

**Affiliations:** 1 Climate Change Research Centre and ARC Centre of Excellence for Climate Extremes, UNSW, Sydney, NSW 2052 Australia; 2 National Computational Infrastructure (NCI), The Australian National University, Canberra, ACT 2601 Australia; 3 Barcelona Supercomputing Center (BSC), Barcelona, Spain; 4 Institució Catalana de Recerca i Estudis Avançats (ICREA), Barcelona, Spain; 5 Centre for Marine Science and Innovation and Australian Centre for Excellence in Antarctic Science, UNSW, Sydney, NSW 2052 Australia

**Keywords:** Atmospheric dynamics, Projection and prediction

## Abstract

Several aspects of regional climate including near-surface temperature and precipitation are predictable on interannual to decadal time scales. Despite indications that some climate states may provide higher predictability than others, previous studies analysing decadal predictions typically sample a variety of initial conditions. Here we assess multi-year predictability conditional on the phase of the El Niño–Southern Oscillation (ENSO) at the time of prediction initialisation. We find that predictions starting with El Niño or La Niña conditions exhibit higher skill in predicting near-surface air temperature and precipitation multiple years in advance, compared to predictions initialised from neutral ENSO conditions. This holds true in idealised prediction experiments with the Community Climate System Model Version 4 and to a lesser extent also real-world predictions using the Community Earth System Model and a multi-model ensemble of hindcasts contributed to the Coupled Model Intercomparison Project Phase 6 Decadal Climate Prediction Project. This enhanced predictability following ENSO events is related to phase transitions as part of the ENSO cycle, and related global teleconnections. Our results indicate that certain initial states provide increased predictability, revealing windows of opportunity for more skillful multi-year predictions.

## Abstract (web summary)

The study identifies windows of opportunity for multi-year climate predictions, depending on the state of ENSO. Predictions started during El Niño and La Niña exhibit higher skill than predictions started during neutral ENSO conditions.

## Introduction

Interannual to decadal climate predictions aim to predict the climate state for the following year and up to 10 years into the future. These decadal predictions are affected by both external forcing and internal climate variability and are performed by initialising a climate model with observations or reanalyses and integrating them for 10 years after initialisation^1,2^. A general warming trend, associated with increasing greenhouse gas forcing, provides some skill for temperature on decadal time scales almost everywhere^3^. Aligning the climate variability by initialising the predictions with the observed climate has been shown to add skill in some regions, including the North Atlantic, the Amundsen Sea, and some land regions including Europe, the Middle East and Africa^2–4^. Even precipitation, which has lower decadal predictability than temperature, exhibits significant forecast skill on multi-year to decadal time scales over some regions, including parts of Europe, Asia, the Sahel, and North America^3,5,6^.

Studies of decadal climate predictability usually quantify skill using predictions started from all initialised states. This approach determines the general level of predictability, but it neglects possible forecasting windows related to more predictable situations that could enable more skilful predictions under certain conditions, as in a Lorenz system^7^. For example, on weather time scales (e.g. hours to days), high-pressure weather systems are usually linked to more predictable weather (i.e. persistent fine conditions) compared to storms within low-pressure systems, that are generally more transient and variable^8^. For conditional predictability on seasonal time scales, forecasts initialised in June show modest skill in predicting sea surface temperature (SST) in the eastern tropical Pacific for up to 6–9 months compared with less skilful forecasts initialised before the boreal spring predictability barrier in April and May^9^. On decadal time scales, increased predictability has been found when initialising from strong Atlantic Meridional Overturning Circulation (AMOC) or Atlantic Ocean Heat Transport (OHT) than from weak AMOC or OHT conditions^10,11^. A recent effort to reconstruct states of the tropical Pacific suggested it exhibits different predictability depending on the climate state at the time of initialisation^12^.

As one of the globally dominant modes of interannual climate variability, El Niño-Southern Oscillation (ENSO) is related to ocean temperature and coupled atmospheric circulation anomalies in the tropical Pacific, affecting many remote parts of the globe via teleconnections^13–17^. ENSO has also recently been shown to contribute to decadal predictability in the South Pacific^18^, and inter-annual rainfall predictions for monsoon regions were shown to be more skilful when ENSO is active^19^. ENSO has also been shown to possibly trigger transitions in the Interdecadal Pacific Oscillation, which could be a source of multi-annual predictability^20^. However, no previous studies have systematically investigated multi-year to decadal predictions starting from different ENSO phases at a global scale. In this study, using perfect-model prediction experiments and retrospective predictions of the observed climate, we investigate multi-year climate predictability and its sensitivity to ENSO phase when the predictions are initialised^21^. We classify the predictions according to ENSO phase at the time when the predictions are initialised with El Niño, La Niña and neutral conditions, and compare the forecast accuracy between these three different phases.

## Results

### Higher multi-year predictability after El Niño and La Niña events

**Fig. 1:**
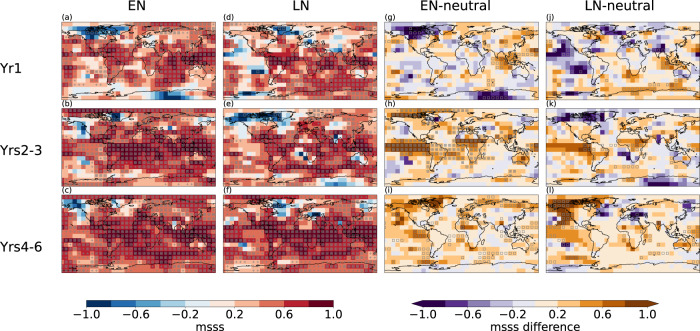
Potential skill dependence on initial El Niño-Southern Oscillation (ENSO) state. The mean squared skill score (MSSS) for the near-surface temperature in the perfect-model predictions for decadal simulations initialised in El Niño (EN; **a**, **b**, **c**), La Niña (LN; **d**, **e**, **f**), and the skill difference between the different groups: El Niño – neutral (**g**, **h**, **i**) and La Niña – neutral (**j**, **k**, **l**). Rows correspond to forecast year 1 (**a**, **d**, **g**, **j**), the average of forecast years 2–3 (**b**, **e**, **h**, **k**), and the average of forecast years 4–6 (**c**, **f**, **i**, **l**). The plus sign stippling indicates grid cells where the skill score or skill differences are significant at the false discovery rate (FDR) being 0.2 (when adjusting for the false discovery rate, see Methods for details). The square symbols indicate grid cells where the skill score or skill differences are significant at the 90% level (outside the 5%–95% confidence interval based on local individual testing with 1000 bootstrap realisations).

The perfect-model predictions show positive potential skill in most regions of the globe for multi-year to decadal predictions of near-surface temperature (Fig. 1); a previous study showed that added skill from initialisation is found primarily in the first two forecast years across the sample of all starting years^21^. Analysing the potential skill for the different groups according to ENSO conditions at the beginning of the predictions, we also find large areas of the globe where the mean squared skill score (MSSS; see Methods) is positive (Fig. 1 for predictions started in El Niño and La Niña conditions, and Supplementary Fig. S1 for predictions started in neutral conditions). In the first year after initialisation, the skill for predictions started in neutral conditions is still high, so that the differences between the different groups are small. For multi-year forecast times beyond the first year, however, substantially larger regions of the globe exhibit skill in predictions starting from El Niño or La Niña conditions, when compared to predictions starting from neutral years. This is particularly the case in the tropical Pacific and tropical Atlantic, as well as over parts of Africa, for the average of forecast years 2–3 (Fig. 1h, k). For the 4–6 year forecasting period, enhanced skill is found over the extratropical North Pacific and parts of the Southern Ocean (Fig. 1i, l).

**Fig. 2:**
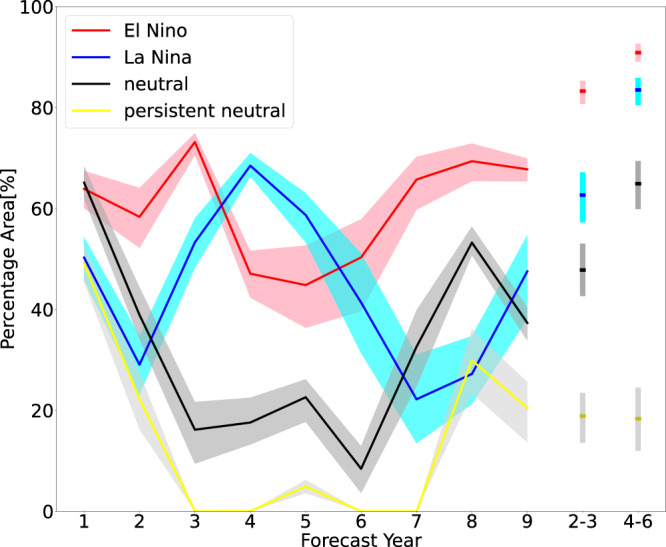
Areas of skill. The percentage of global areas (10° × 10° grid cells weighted by cos(latitude)) where the perfect-model predictions exhibit significant skill (the false discovery rate (FDR) is 0.2 when adjusting for the false discovery rate as indicated by the ‘+’ sign stippling in Fig. 1) for predictions initialised in El Niño (red), La Niña (blue), neutral (black), and persistent neutral (yellow) conditions. The shading around each line represents the 5%–95% confidence interval based on 1000 bootstrap realisations (with replacement, see Methods).

Figure 2 shows about half of the global area exhibits significant skill in predicting annual averages of near-surface temperature at 3, 4, 5 and 6 years in advance when starting from El Niño and La Niña events. In contrast, <20% of the global area has significant skill when starting from neutral conditions. For multi-year averages, the skilful areas are even larger: for example, more than 80% of the area exhibits significant skill after El Niño conditions for the 2–3 year prediction period, versus ~50% for predictions initialised in neutral years. The areas of significant prediction skill are ~90% (after El Niño years) versus 60% (neutral years) for the 4–6 year forecast period. In addition, the curve of classical neutral conditions (i.e. defined as all years that are not classified as El Niño or La Niña) is always above the curve of persistent neutral conditions (defined as years when there are no excursions to La Niña or El Niño conditions (see Methods); grey line in Fig. 2). This indicates predictability is even lower when initialising predictions from persistent neutral conditions during November, December and January (NDJ) and when average SST anomalies over these 3 months are smaller. Based on the MSSS differences (the two right-most columns in Fig. 1), potential skill is higher after El Niño conditions than after neutral conditions in more than 30% (forecast years 2–3) and more than 20% (forecast years 4–6) of the globe (Supplementary Fig. S2). After La Niña conditions, about 10% of the globe shows positive MSSS differences, whereas area fractions where MSSS is higher after neutral conditions are very small.

**Fig. 3:**
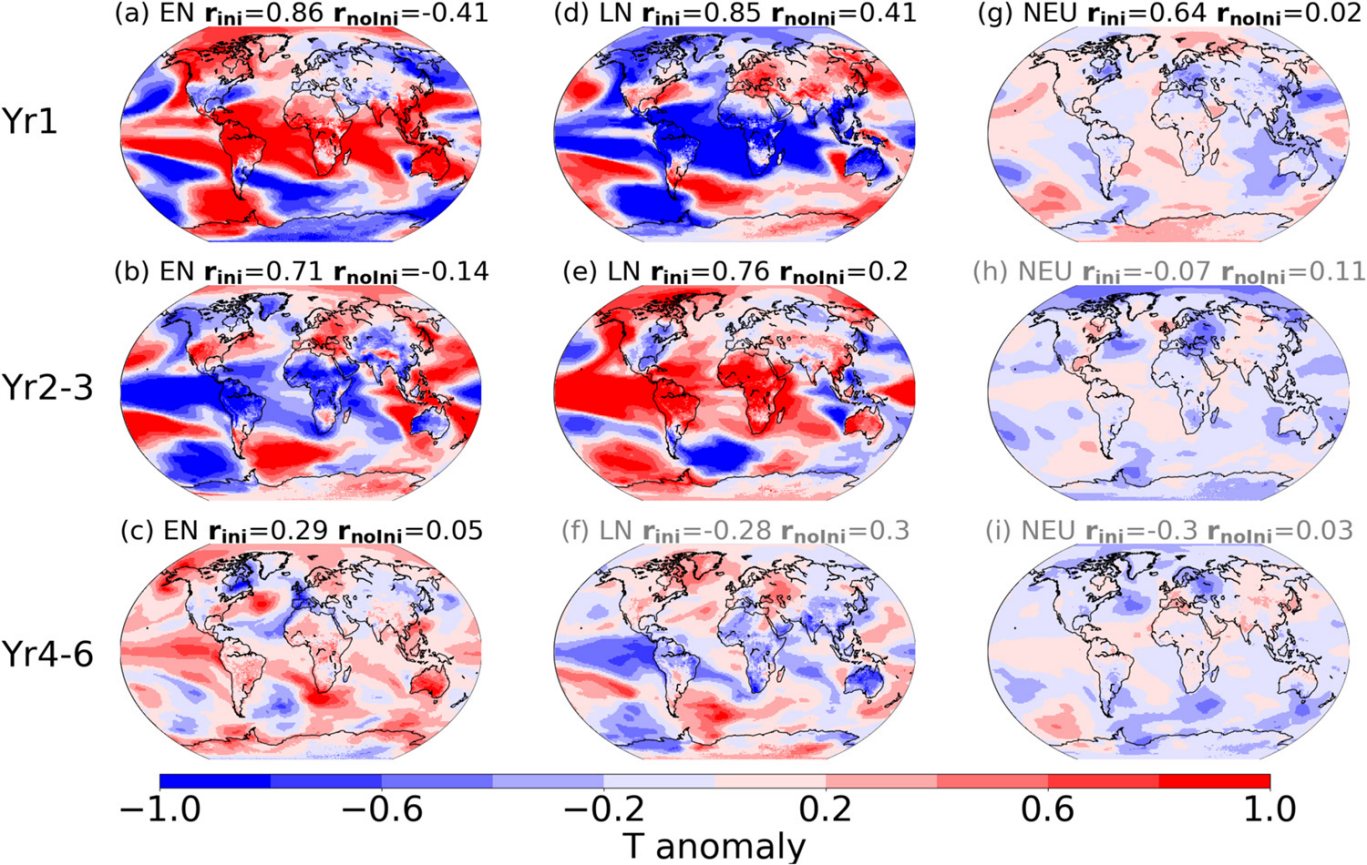
Agreement of local temperature anomalies. Composite maps of normalised temperature anomalies (after detrending; refer to Methods for details of normalisation and detrending) from perfect-model decadal simulations started from El Niño (EN; **a**, **b**, **c**), La Niña (LN; **d**, **e**, **f**) and neutral (NEU; **g**, **h**, **i**) conditions for forecast years 1 (**a**, **d**, **g**), 2–3 (**b**, **e**, **h**) and 4–6 (**c**, **f**, **i**). The pattern correlations (weighted by grid cell areas) of the initialised (r_ini_) and uninitialized (r_noIni_) perfect-model predictions compared to the reference simulation are shown above each panel. Subplot titles are in black when r_ini_ is significantly greater than r_noIni_ (exceding the one-sided 95% confidence interval, based on 1000 bootstrap realisations), whereas grey subplot titles show non-significant difference between r_ini_ and r_noIni_.

Composite maps of predictions initialised with El Niño and La Niña conditions show distinct temperature anomaly patterns reflecting the ENSO phase and related teleconnections during the first forecast year (Fig. 3). These include warm anomalies in most tropical regions (extending to higher latitudes along the west coasts of North and South America) and cold anomalies in the extratropical North and South Pacific in the first year after El Niño events, and largely opposite sign anomalies after La Niña events. In contrast, local mean anomalies are small in predictions initialised from neutral conditions. The average temperature anomaly over forecast years 2 and 3 shows anomaly patterns of mostly opposite sign compared to the first forecast year, suggesting that the climate in these simulations exhibits a tendency to evolve to the opposite ENSO phase of the initial state in the second or third year after initialisation. The average over forecast years 4–6 again shows similar anomalies to the first year, particularly across the tropical Pacific, indicating that the climate system tends to evolve back towards the initial ENSO phase at some point during this period, affecting the 3-year average (over forecast years 4, 5, and 6) accordingly. These tendencies for the Pacific to evolve into the opposite phase of ENSO over a 2-3 year time scale are not a given of course; the enhanced predictability is merely indicative of likelihoods given the initial climate state in the forecast.

The global anomaly patterns from the initialised perfect-model predictions closely resemble the patterns derived from the reference run of the perfect-model predictions (compare Fig. 3 and Supplementary Fig. S3) for up to 3 years after El Niño and La Niña conditions (pattern correlations >0.7). In particular, pattern correlations between the predicted anomalies and the reference run anomalies are higher than the pattern correlations of the historical (uninitialized) simulations with the reference run (maps are shown in Supplementary Fig. S4; pattern correlations for both initialised and uninitialized predictions are shown above each map in Fig. 3). This is true for the 2–3 year forecast period after both El Niño and La Niña events, and for the 4–6 year forecast period after El Niño, indicating added skill from initialisation at the global scale. In contrast, pattern correlations are low, and anomalies comparably smaller for predictions initialised from neutral conditions. Further, when starting from neutral conditions, pattern correlations show no additional skill from initialisation beyond the first forecast year. Overall similar results are also found for precipitation anomaly patterns (Fig. S5), indicating enhanced precipitation predictability and added value from initialisation for multiple years after El Niño and La Niña events. Note that the different rows in Figs. 3, S3 and S4 average over a different number of years (i.e. 1, 2 and 3 years), and this averaging will lead to different spatial correlations across the different rows. The key information for this comparison is that the initialised predictions show a more similar pattern to the reference run than the uninitialized predictions.

### ENSO cycle determines the conditional predictability

The added skill in the tropical Pacific may be explained by ENSO periodicity. For example, in the tropical Pacific region where SST variations related to ENSO are strongest, predictions starting from either El Niño or La Niña are more skilful than those starting from neutral conditions (Fig. 1h, i, k, l). This could be indicative of the potential skill in capturing ENSO variations subsequent to El Niño/La Niña events^22–24^. The model exhibits an ENSO periodicity of 3–6 years, consistent with observations (Fig. S6). Therefore, it seems plausible that the enhanced predictability after ENSO peak phases (i.e. after either El Niño or La Niña events) could be related to the dominant periodicity of the ENSO cycle as seen in the tendency for evolution to the opposite ENSO phase after 2–3 years and back to initial phase after 4–6 years (Fig. 3). This hypothesis is confirmed by analysing the potential skill in predicting ENSO itself, measured e.g. by the Niño3.4 index (Fig. S7). Predictions started from El Niño and La Niña states are more skilful in predicting the Niño3.4 index compared to predictions started in neutral conditions, or skill calculated based on all annual initialisations for the forecast years 2–3 and forecast years 4–6 (although uncertainty ranges based on bootstrapping the relatively small samples are large). In forecast year 1 the potential skill of predictions started in neutral conditions (and in particular persistent neutral conditions) is very high. This is likely due to the relatively small temperature variations in such cases when no ENSO phase transition happens in the months following initialisation, and SST anomalies remain relatively small.

The predictable tropical Pacific conditions also appear to result in higher potential skill in other remote locations due to both inter-basin and tropical-extratropical teleconnections. For example, tropical Pacific SST anomalies related to ENSO cycles teleconnect to both high southern and northern latitudes, and the tropical Pacific is known to interact with both the tropical Indian and Atlantic Oceans via adjustments in the Walker circulation^25–29^. In particular, the simulated anomaly in the tropical Pacific region co-exists with same-sign high-latitude anomalies over North America, the Arctic, and the Amundsen Sea regions for both temperature (Fig. 3) and precipitation (Fig. S5). Such correspondence of regional anomalies is found during e.g. 1, 2–3 and 4–6 years after El Niño, years 1 and 2–3 after La Niña in Fig. 3; and years 1 and 2–3 after El Niño and La Niña in Fig. S5).

From an oscillator point of view, peak phases of the ENSO oscillation (i.e. El Niño and La Niña events) are most likely followed by a transition towards the opposite phase, except in a few cases when these events (in particular La Niña) can endure for a second year^30^. In contrast, after neutral start years, there is less predictive skill as the system could evolve towards either El Niño or La Niña, or indeed remain neutral. In order to compare transition probabilities based on different initial conditions in our perfect-model experiment, we investigate the average transition probabilities over all forecasting members of the El Niño, La Niña and neutral starting points respectively during different forecasting times; namely 1, 2–3 and 4–6 years (Table S2). This analysis confirms that El Niño start years are mostly followed by neutral or La Niña years in the first year after initialisation; while La Niña start years are more likely followed by neutral or El Niño years. The simulations have an increased probability to transition into the opposite phase during forecast year 2 or 3 after initialisation compared to transitioning into the initial phase. In particular, simulations started in El Niño conditions have a probability to transition into La Niña at some point during forecast year 2 or 3 of 32%, whereas only 19% transition back to El Niño, and simulations started in La Niña conditions have a probability of 37% to transition into El Niño during forecast year 2 or 3, but only 22% probability to transition back into La Niña. During forecast years 4, 5 or 6 there is a slightly increased probability for the simulations to transition back into the initial state (i.e. after El Niño to El Niño and after La Niña to La Niña) than into the opposite phase. In contrast, for the neutral start years, the probability of transition to El Niño is comparable to La Niña for the 2–3 year and 4–6 year forecast periods. Inconsistent variations (i.e. of opposite sign) across ensemble members following different neutral initial states tend to cancel one another out, so that average anomalies after neutral conditions are relatively small (Figs. 3 and S6).

The interannual phase variations of ENSO are driven by the recharge and discharge of heat along the tropical Pacific Ocean over a dominant 3-6 year time scale^31^. Capturing the ENSO phase in the initial state appears to be the primary agent for improved multi-year predictability, with the reliability of these predictions dependent on the representation of the dominant time scales of ENSO variability (e.g. the power spectral analysis of Fig. S6). In the context of this study, when initialising from either El Niño or La Niña phases, the non-equilibrium component between the zonal mean equatorial thermocline depth and wind stress of the recharge-discharge oscillator is included in the initialisation. Initialising from neutral states, however, does not guarantee the inclusion of the imbalance between the subsurface ocean temperature and atmospheric winds, as the SST, sea level pressure, sea surface height and surface wind anomalies are very weak. Indeed, we found the difference in prediction skill between ENSO peak phases and neutral states to be more pronounced for persistent neutral states (i.e. those not exceeding the El Niño or La Niña threshold in any single month during NDJ) than for the classical neutral states (i.e. those not identified as El Niño or La Niña). That is, without any (even brief) excursion to El Niño or La Niña states, as in the persistent neutral states, there may be less non-equilibrium information embedded in the initialisation. This finding suggests that, in addition to the simulated ENSO cycle, stronger initial SST anomalies in the tropical Pacific may lead to enhanced skill. This is plausible when a stronger non-equilibrium component of the recharge-discharge oscillator is related to a lower error growth rate and a better-defined attractor evolution, compared to situations with smaller SST anomalies and related non-equilibrium components.

### Windows of opportunity in real-world predictions

**Fig. 4:**
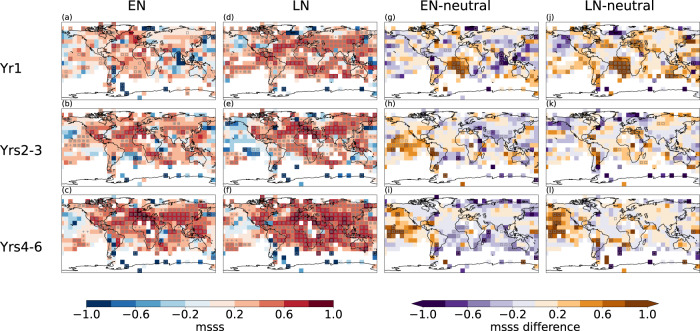
Skill dependence on initial El Niño-Southern Oscillation (ENSO) status in decadal hindcasts of the real-world climate. The mean squared skill score (MSSS) of the near-surface temperature for the real-world hindcasts provided by the Community Earth System Model decadal prediction large ensemble (CESM-DPLE) simulations^5^ initialised in El Niño (EN; **a**–**c**), La Niña (LN; **d**–**f**), and the skill difference between the different groups: El Niño – neutral (**g**–**i**) and La Niña – neutral (**j**–**l**). Rows correspond to forecast year 1 (**a**, **d**, **g**, **j**), the average of forecast years 2–3 (**b**, **e**, **h**, **k**), and the average of forecast years 4–6 (**c**, **f**, **i**, **l**). The plus sign stippling indicates grid cells where the skill score or skill differences are significant at the false discovery rate (FDR) being 0.2 (when adjusting for the false discovery rate, see Methods for details). The square symbols indicate grid cells where the skill score or skill differences are significant at the 90% level (outside the 5%–95% confidence interval based on local individual testing with 1000 bootstrap realisations). HardCRUT4-median is used as the observation ref. ^59^.

Both the model and the observational data indicate a peak in the spectral intensity of Niño3.4 SST at around 3–6 years (Fig. S6). This indicates similar ENSO periodicity in the model and real-world, consistent also with previous evaluations that showed realistic ENSO characteristics of the model version used here^32,33^, and suggests that we may also achieve enhanced skill following ENSO events in real-world predictions. In fact, the real-world hindcasts also show enhanced skill for predictions starting from El Niño and La Niña compared to neutral conditions for the forecast periods 2–3 and 4–6 years, in particular in the Pacific Ocean (Fig. 4 for predictions started in El Niño and La Niña conditions, and Supplementary Fig. S8 for predictions started in neutral conditions), while some negative MSSS differences are found in other regions outside the Pacific. Despite the generally lower skill compared to the perfect-model predictions, there is positive MSSS in the central Pacific in forecast years 2–3 after El Niño events, whereas skill in this region is negative after neutral initial conditions—pointing to a window of opportunity also in real-world predictions. As the enhanced multi-annual skill after El Niño and La Niña conditions is spatially more limited to the Pacific, also the global area fraction with positive skill differences is substantially smaller compared to the perfect-model predictions (Supplementary Fig S9).

These results based on the CESM model are largely consistent with the retrospective predictions provided within the Coupled Model Intercomparison Project phase 6 (CMIP6) Decadal Climate Prediction Project (DCPP)^34^. The multi-model ensemble of decadal hindcasts (which does not include the CESM; see the list of DCPP models in Supplementary Table S4) also shows enhanced skill in particular in parts of the Pacific in predictions initialised in El Niño and La Niña conditions compared to predictions initialised in neutral conditions (Supplementary Fig. S10)^35^. Similar to the real-world predictions with the CESM model, this enhanced skill is not transferred to other regions outside the Pacific (in particular for multi-annual timescale predictions), possibly due to inconsistent representation of relevant teleconnections in the different models^36–38^. Still these results indicate windows of opportunity for multi-annual predictions related to the ENSO state for large parts of the Pacific Ocean (including SSTs in the NINO3.4 region, Supplementary Fig. S11), and improved models may in the future transfer the enhanced skill also to other regions.

Given the similar ENSO periodicity between the CESM model and observations and the generally low skill over the Pacific region in the real-world predictions, it is likely that this overall lower skill in the real-world hindcasts compared to the perfect-model is related to non-ideal initialisation of the hindcasts and related adjustments^5^, unresolved ocean dynamics, or inconsistent representation of long-term trends between model and observations in the Pacific^5,21,39–41^. Therefore, real-world predictions may be improved with improved initialisation, in terms of both observational coverage and model-observation consistency, to reduce both the uncertainty of the observed initial state and the shock and drift of the simulations after initialisation.

ENSO transition probabilities calculated from observations also indicate an increased chance for the opposite phase to occur 2 or 3 years in particular after La Niña events (Table S3), consistent with the transitions in the model. No clear probability differences are found for the pooling of forecast years 4, 5, 6. However, there is an increased probability of La Niña events compared to El Niño 5, 6 or 7 years after La Niña events—pointing to possibly slightly longer ENSO cyclicity in observations compared to the model.

## Discussion

We have demonstrated that multi-year predictions started from El Niño and La Niña conditions show significantly higher potential and actual skill than predictions started from neutral conditions, based on perfect-model prediction experiments with the Community Climate System Model Version 4 and real-world hindcasts with CESM and a CMIP6 multi-model ensemble. This enhanced skill seems to be related to the simulated ENSO cycles and related teleconnections after ENSO peak phases, while after neutral conditions a cancellation of different phase transitions reduces predictability. The simulated ENSO cycles imply some multi-year predictability of ENSO events, consistent with the finding that some ENSO events can be predicted as much as four years ahead in real-world hindcasts^42^. Our results also suggest that enhanced predictive skill related to ENSO is potentially achievable if remaining shortcomings related to the initialisation of the model predictions could be improved, as well as the representation of relevant dynamics in the model. Despite the generally lower skill in comparison to the perfect-model case, there are instances where skill over parts of the Pacific is positive after El Niño or La Niña conditions and negative after neutral conditions—hinting to windows of opportunity also in the real-world predictions. Even though the actual skill is low, this gives hope that if the general weakness of low skill in the Pacific can be improved in decadal prediction systems, the conditional predictability on ENSO may provide more skilful ‘windows of opportunity’.

Temporal variations in predictability that indicate ‘windows of forecast opportunity’ have previously been noted in sub-seasonal to seasonal predictions, where certain regimes and persistent anomalies can provide added information above the weather noise^43^. For example, El Niño or La Niña events can provide forecasting windows on seasonal time scales^43^. Furthermore, ENSO predictability can undergo pronounced multi-decadal variations and the multi-decadal variability in the strength of ENSO teleconnections affects the predictive skill of extratropical teleconnections^44,45^. Our work indicates that such windows of opportunity also exist for multi-annual predictions, where predictions starting from El Niño and La Niña states exhibit larger predictable regions compared to those starting from neutral years for forecast times longer than one year. Our study suggests that conditionally enhanced prediction skill from specific climate states can yield improved predictions at certain points in time. This paves the way for climate services to provide more credible multi-year predictions at certain times with specific climate states.

## Methods

We investigate multi-year predictability conditional on the ENSO phase at the time of initialisation in both perfect-model predictions and hindcasts of the real-world climate. To study the perfect-model predictability, we performed experiments with the Community Earth System Model (CESM) version 1.0.5, a fully coupled model comprising atmospheric, oceanic, land and sea ice models. We used a configuration of the model that corresponds to the Community Climate System Model Version 4 (CCSM4), i.e. with the atmospheric component Community Atmosphere Model (CAM4) (at a horizontal resolution of 1.25° × 0.9°, with 26 vertical layers)^46^; the ocean component Parallel Ocean Program version 2 (at a 1 degree horizontal resolution and 60 vertical levels on a displaced-pole grid)^47^; the land component Community Land Model version 4 (run in the atmospheric grid)^48^; and the sea ice component Community Ice Code version 4 (run in the ocean grid)^49^. This is the same model configuration used for the CCSM4 contributions to Coupled Model Intercomparison Project phase 5 (CMIP5) historical and Representative Concentration Pathway (RCP) scenarios from which we are using transient historical simulations as uninitialized reference. For simplicity, we therefore refer to our perfect-model predictability experiments as CCSM4.

The first set of simulations includes perfect-model predictions that were performed by initialising an ensemble of decadal simulations from annual restart files of a transient historical simulation^21^. The term ‘perfect-model predictions’ refers to experiments in which a model is used to predict the climate evolution of that same model as opposed to a real-world climate, meaning that the model is a physically perfect representation of the reference it is aiming to predict, meaning that predictability estimates are thus not affected by inconsistencies between model and real-world climate. These initialised perfect-model predictions include five ensemble members initialised from every January 1st of the 46 initialisation years from 1961 to 2006. The ensemble members were generated by adding small Gaussian perturbations (order of 10^−5^K) to the atmospheric temperatures, with all other variables being identical, to simulate the case of perfectly known initial conditions^21^. The potential skill is then evaluated by quantifying how well these decadal simulations predict the transient reference simulation from which they were initialised. We compare the potential skill of the perfect-model predictions against the second set of uninitialized, transient climate simulations that contributed to CMIP5. These simulations are performed with the same model as the reference simulation they are used to predict, so we refer to these as uninitialized perfect-model predictions. Same as the initialised perfect-model predictions and the transiently forced reference run, these simulations use historical forcing until 2005 and then follow the RCP4.5 scenario from 2006 to 2015. For these uninitialized perfect-model predictions, we also use five ensemble members (started from different states of a pre-industrial control simulation to sample a wider range of internal variability), available within CMIP5, matching the ensemble size of the initialised perfect-model predictions^21^. Note that the ENSO periodicity was overly biennial in older versions of the CCSM model, e.g. CCSM3, but these issues have been much improved in the CCSM4 version which shows a typical ENSO periodicity of 3-6 years, consistent with observations as seen in the power spectrum shown in Fig. S6^32,33,50^. CCSM4, CESM1 and the newer version CESM2 were shown to simulate ENSO characteristics such as periodicity and transitions reasonably similar to observations^33^.

For real-world predictions, we utilise the CESM decadal prediction large ensemble (CESM-DPLE), which has 40 ensemble members each initialised on November 1st from 1954 to 2015 that are integrated for 122 months^5^. Note the atmospheric component of CCSM4 that we use for the perfect-model experiment is the Community Atmosphere Model 4 (CAM4)^46^. CESM-DPLE used in the real-world hindcast utilises the Community Atmosphere Model 5 (CAM5) as its atmospheric component^5^. As we do not directly compare perfect-model predictions and real-world hindcasts, our conclusions do not require identical model versions or configurations for these different experiments.

Classifying the starting years of the perfect model and the real-world predictions from 1961 to 2006 according to ENSO phase, the basic methodology of the analyses in this study is to analyse skill measures and composites for these different ENSO groups of predictions. Detrended 5-month running means of monthly SSTs from the Nino3.4 region are used to estimate the Nino3.4 index. The detrending is done by subtracting the 30-year moving climatology preceding the year under consideration. This Nino3.4 index is used to classify each of the starting years as either El Niño, La Niña or neutral. El Niño start years are when the Nino3.4 index exceeds the upper quartile threshold (computed over the 1960–2015 period) from November to January (NDJ). La Niña start years are when the Nino3.4 index is below the lowest quartile. All remaining years are classified as neutral (this is referred to as the ‘classical’ neutral year definition in this paper). However, in the conventional neutral year definition (i.e. all years that are not classified as El Niño or La Niña), neutral years are not always characterised by persistent neutral conditions over all the 3 months (NDJ), because the Nino3.4 Index can exceed the quartile thresholds for 1 or 2 months. Therefore, we also consider a second neutral year classification (referred to as persistent neutral), which only includes the years when all 3 months (i.e. November, December and January) exhibit neutral conditions (i.e. excluding those years with intermittent positive or negative conditions during NDJ). The classification of starting years for the perfect-model predictions and real-world predictions are shown in Table S1.

While this ENSO classification includes some information from after the initialisation of the predictions (i.e. January SST, the first forecast month of the perfect-model predictions), this is still useful for interannual or multi-year predictions that target forecast times several years after initialisation. January is included in the classification to consider the entire season when ENSO events typically peak. For classifying ENSO in the real-world hindcasts, which are initialised on November 1st each year (i.e. 2 months earlier than the perfect model), we use the same months (November, December and January, i.e. the first three forecast months of the hindcasts). This classification therefore matches the perfect-model decadal prediction and the ENSO peak period, keeping the definitions consistent between the perfect-model and the real-world predictions.

To examine decadal prediction accuracy, we use the mean squared skill score (MSSS) as the primary deterministic verification metric and follow the framework for evaluating decadal predictions^14,51–54^. MSSS can be decomposed as a function of the hindcasts, climatological forecast and observations when the climatological forecast is used as the reference, namely:

1 $${{{{{\rm{MSSS}}}}}}\left(H,\bar{O,}\,O\right)={r}_{{HO}}^{2}-{\left[{r}_{{HO}}-\frac{{S}_{H}}{{S}_{O}}\right]}^{2}-{\left[\frac{\bar{H}-\bar{O}}{{S}_{O}}\right]}^{2}$$

where ${r}_{{HO}}$ represents the sample correlation between the hindcasts (*H*) and the observations (*O*), ${{S}_{H}}^{2}$ is the sample variance of the ensemble mean hindcasts, *S*_*O*_^*2*^ denotes the sample variance of the observations, $\bar{O}={\sum }_{j=1}^{n}{O}_{j}$ is the climatological forecast (of which ${O}_{j}$ represents the observations, or perfect-model reference respectively, over *j* = 1,...,*n* starting times), and $\bar{H}$ is the mean hindcast.

The evaluation process is implemented in the Miklip evaluation system^55^. The temperature and precipitation data are remapped to a 10° × 10° grid prior to calculating the skill measures to reduce small‐scale noise effects^51^. To account for model drift (which is a pertinent issue in the initialised real-world predictions), anomalies are calculated from lead-time dependent climatologies. We use 1000 bootstrap realisations (with replacement) to test the significance level of MSSS and skill differences between the different ENSO initialisations and estimate the *p* values of the local grid points. Each bootstrap realisation differs in a combination of starting years, and 5-year blocks are maintained to preserve temporal autocorrelation related to low‐frequency variability in resampling^51^. We further assess field significance and control for the false discovery rate (FDR), thereby minimising potential overinterpretation related to multiple significance testing^56^. For a local grid point to pass the adjusted significance test with controlled *P*_*FDR*_, its *p* value (p(i) as shown in Eq. 2) has to be smaller than the *P*_*FDR*_ calculated from the following equation:

2 $${P}_{{FDR}}=\max [p(i):p(i)*(i/N)*{\alpha }_{{FDR}}](i=1,\ldots,N)$$

where *N* is the total number of grid points and *i* is the rank of sorted *p* values. Significance is assessed at ${\alpha }_{{FDR}}$ = 0.2 in this case. For fields with moderate to high auto-correlation ${\alpha }_{{FDR}}$ = 0.2 corresponds to the significance level of ${\alpha }_{{global}}$ = 0.1^56^.

To estimate the uncertainty of global area fractions with significant skill (e.g. Fig. 2), we also use the 1000 bootstrap realisations described above to estimate the significance of skill values. To obtain a range of *p* values for each grid cell (based on which then areas with skill can be calculated), we subsample 1000 times 100 of these realisations (with replacement).

To avoid some systematic differences in temperature caused by the uneven distribution of La Niña or El Niño years over the investigation period (note our historical reference run used to initialise the perfect-model predictions exhibits the majority of El Niño events in the later, warmer, part of the investigation period), we remove the forced signal in the temperature and precipitation fields before compositing them. These composite maps (e.g. Fig. 3) therefore demonstrate common features of internal variability. To do this, we use the ensemble mean of six CCSM4 historical simulations from CMIP5 together with our own historical simulation (i.e. seven members in total) to determine the forced response at each grid point for each year. We also apply an 11-year running average to the ensemble to remove responses to other short-term forcing, such as volcanoes^57^. We then deduct the smoothed ensemble mean from the reference run and the decadal predictions to obtain the detrended data. Subsequently, El Niño, La Niña and neutral group anomaly composite maps are calculated by subtracting the composite of all years from each group. For both the temperature and precipitation composite maps, we normalise the anomalies by dividing them by the standard deviation of the temperature and precipitation time series with annual or multi-annual (i.e. for forecast years 2–3 and 4–6) averages on each grid point in order to ensure anomalies in the composite maps are comparable across regions (e.g. tropics where temperature variability is usually small versus high latitudes where variability is larger).

### Reporting summary

Further information on research design is available in the Nature Portfolio Reporting Summary linked to this article.

### Supplementary information

Supplementary Information

Reporting Summary

## Data Availability

The perfect-model prediction output data used in this study are available in NCI Australia data collections^58^. The CESM-DPLE data used in this study are available for downloading through https://www.cesm.ucar.edu/community-projects/dple. The temperature observation data used in this study are accessible at https://www.metoffice.gov.uk/hadobs/hadcrut4/; https://www.metoffice.gov.uk/hadobs/hadisst/; https://data.giss.nasa.gov/gistemp/; https://psl.noaa.gov/data/gridded/data.mlost.html.

## Code availability

The tool that calculates the Mean Squared Skill Score (MSSS) is available through Github at https://github.com/illing2005/murcss.

## References

[1] Meehl GA (2009). Decadal Prediction. Bull. Am. Meteorol. Soc..

[2] Meehl GA (2014). Decadal Climate Prediction: An Update from the Trenches. Bull. Am. Meteorol. Soc..

[3] Smith, D. M. et al. Robust skill of decadal climate predictions. *Npj Clim. Atmos. Sci.* **2**, 10.1038/s41612-019-0071-y (2019).

[4] Doblas-Reyes, F. J. et al. Initialized near-term regional climate change prediction. *Nat. Commun.* **4**, 10.1038/ncomms2704 (2013). 10.1038/ncomms2704 PMC3644073 23591882

[5] Yeager SG (2018). Predicting Near-Term Changes in the Earth System: A Large Ensemble of Initialized Decadal Prediction Simulations Using the Community Earth System Model. Bull. Am. Meteorol. Soc..

[6] Solaraju-Murali, B., Caron, L.-P., Gonzalez-Reviriego, N. & Doblas-Reyes, F. J. Multi-year prediction of European summer drought conditions for the agricultural sector. *Environ. Res. Lett.* **14**, 10.1088/1748-9326/ab5043 (2019).

[7] Lorenz EN (1969). Atmospheric predictability as revealed by naturally occurring analogues. J. Atmos. Sci..

[8] Wallace, J. M. & Hobbs, P. V. *Atmospheric science: an introductory survey*. 2nd edn, (Amsterdam; Boston: Elsevier Academic Press, 2006).

[9] Tippett, M. K., L’Heureux, M. L., Becker, E. J. & Kumar, A. Excessive Momentum and False Alarms in Late-Spring ENSO Forecasts. *Geophys. Res. Lett.* **47**, 10.1029/2020GL087008 (2020).

[10] Collins M, Botzet M, Carril AF, Drange H (2006). Interannual to Decadal Climate Predictability in the North Atlantic: A Multimodel-Ensemble Study. J. Clim..

[11] Borchert LF, Müller WA, Baehr J (2018). Atlantic Ocean Heat Transport Influences Interannual-to-Decadal Surface Temperature Predictability in the North Atlantic Region. J. Clim..

[12] Ramesh N, Cane MA (2019). The Predictability of Tropical Pacific Decadal Variability: Insights from Attractor Reconstruction. J. Atmos. Sci..

[13] McPhaden MJ, Zebiak SE, Glantz MH (2006). ENSO as an Integrating Concept in Earth. Sci. Sci..

[14] Stan C (2017). Review of Tropical-Extratropical Teleconnections on Intraseasonal Time Scales. Rev. Geophys..

[15] Trenberth KE (1998). Progress during TOGA in understanding and modeling global teleconnections associated with tropical sea surface temperatures. J. Geophys. Res-Oceans.

[16] Li, Q. & England, M. H. Tropical Indo‐Pacific Teleconnections to Southern Ocean Mixed Layer Variability. *Geophys. Res. Lett.* **47**, 10.1029/2020gl088466 (2020).

[17] Turner J, Phillips T, Hosking JS, Marshall GJ, Orr A (2013). The Amundsen Sea low. Int. J. Climatol..

[18] Saurral RI, García-Serrano J, Doblas-Reyes FJ, Díaz LB, Vera CS (2020). Decadal predictability and prediction skill of sea surface temperatures in the South Pacific region. Clim. Dyn..

[19] Dunstone, N. et al. Skilful interannual climate prediction from two large initialised model ensembles. *Environ. Res. Lett.* **15**, 10.1088/1748-9326/ab9f7d (2020).

[20] Meehl GA, Teng H, Capotondi A, Hu A (2021). The role of interannual ENSO events in decadal timescale transitions of the Interdecadal Pacific Oscillation. Clim. Dyn..

[21] Liu Y (2019). A Framework to Determine the Limits of Achievable Skill for Interannual to Decadal Climate Predictions. J. Geophys. Res. Atmos..

[22] MacMynowski DG, Tziperman E (2008). Factors Affecting ENSO’s Period. J. Atmos. Sci..

[23] Santoso A, McPhaden MJ, Cai W (2017). The Defining Characteristics of ENSO Extremes and the Strong 2015/2016 El Niño. Rev. Geophys..

[24] Timmermann A (2018). El Nino-Southern Oscillation complexity. Nature.

[25] McGregor S (2014). Recent Walker circulation strengthening and Pacific cooling amplified by Atlantic warming. Nat. Clim. Change.

[26] Cai W (2019). Pantropical climate interactions. Science.

[27] Meehl, G. A., Chung, C. T. Y., Arblaster, J. M., Holland, M. M. & Bitz, C. M. Tropical Decadal Variability and the Rate of Arctic Sea Ice Decrease. *Geophys. Res. Lett.* **45**, 10.1029/2018gl079989 (2018).

[28] Purich A (2016). Tropical Pacific SST Drivers of Recent Antarctic Sea Ice Trends. J. Clim..

[29] Turner J (2004). The El Niño–southern oscillation and Antarctica. Int. J. Climatol..

[30] DiNezio PN (2017). A 2 Year Forecast for a 60–80% Chance of La Niña in 2017–2018. Geophys. Res. Lett..

[31] Jin FF (1997). An equatorial ocean recharge paradigm for ENSO .1. Conceptual model. J. Atmos. Sci..

[32] Deser C (2012). ENSO and Pacific Decadal Variability in the Community Climate System Model Version 4. J. Clim..

[33] Capotondi, A., Deser, C., Phillips, A. S., Okumura, Y. & Larson, S. M. ENSO and Pacific Decadal Variability in the Community Earth System Model Version 2. *J. Adv. Model Earth Sy.* **12**, 10.1029/2019ms002022 (2020).

[34] Boer GJ (2016). The Decadal Climate Prediction Project (DCPP) contribution to CMIP6. Geosci. Model Dev..

[35] Delgado-Torres C (2022). Multi-Model Forecast Quality Assessment of CMIP6 Decadal Predictions. J. Clim..

[36] Roy I, Gagnon AS, Siingh D (2018). Evaluating ENSO teleconnections using observations and CMIP5 models. Theor. Appl. Climatol..

[37] Ferrett S, Collins M, Ren H-L, Wu B, Zhou T (2020). The Role of Tropical Mean-State Biases in Modeled Winter Northern Hemisphere El Niño Teleconnections. J. Clim..

[38] Garcia-Villada LP, Donat MG, Angélil O, Taschetto AS (2020). Temperature and precipitation responses to El Niño-Southern Oscillation in a hierarchy of datasets with different levels of observational constraints. Clim. Dyn..

[39] Bilbao R (2021). Assessment of a full-field initialized decadal climate prediction system with the CMIP6 version of EC-Earth. Earth Syst. Dynam..

[40] Teng H, Meehl GA, Branstator G, Yeager S, Karspeck A (2017). Initialization Shock in CCSM4 Decadal Prediction Experiments. Glob. Chang. Mag..

[41] Karspeck A, Yeager S, Danabasoglu G, Teng H (2014). An evaluation of experimental decadal predictions using CCSM4. Clim. Dyn..

[42] Gonzalez PLM, Goddard L (2016). Long-lead ENSO predictability from CMIP5 decadal hindcasts. Clim. Dyn..

[43] Mariotti A (2020). Windows of Opportunity for Skillful Forecasts Subseasonal to Seasonal and Beyond. Bull. Am. Meteorol. Soc..

[44] O’Reilly CH (2018). Interdecadal variability of the ENSO teleconnection to the wintertime North Pacific. Clim. Dyn..

[45] Weisheimer, A. et al. Seasonal Forecasts of the Twentieth Century. *Bull. Am. Meteorol. Soc.* **101**, E1413–E1426 10.1175/bams-d-19-0019.1 (2020).

[46] Neale RB (2013). The Mean Climate of the Community Atmosphere Model (CAM4) in Forced SST and Fully Coupled Experiments. J. Clim..

[47] Smith, R. D. et al. The Parallel Ocean Program (POP) reference manual. *Los Alamos National Laboratory Tech.* Rep. LAUR‐10‐01853, 1–140 (2010).

[48] Lawrence, D. M. et al. Parameterization improvements and functional and structural advances in Version 4 of the Community Land Model. *J. Adv. Model Earth Sy*. **3**, 1–27 (2011).

[49] Hunke, E. & Lipscomb, W. CICE: The Los Alamos sea ice model documentation and software user’s manual version 4.0 LA-CC-06-012, *Los Alamos National Laboratory Tech*. Rep*.* LA-CC-06-012, 1–73 (2008).

[50] Deser C, Capotondi A, Saravanan R, Phillips AS (2006). Tropical pacific and Atlantic climate variability in CCSM3. J. Clim..

[51] Goddard L (2013). A verification framework for interannual-to-decadal predictions experiments. Clim. Dyn..

[52] Murphy AHSkill (1988). Scores Based on the Mean-Square Error and Their Relationships to the Correlation-Coefficient. Mon. Weather Rev..

[53] Murphy AH, Epstein ES (1989). Skill Scores and Correlation-Coefficients in Model Verification. Mon. Weather Rev..

[54] Kadow C (2016). Evaluation of forecasts by accuracy and spread in the MiKlip decadal climate prediction system. Meteorologische Z..

[55] Iling, S., Kadow, C., Kunst, O. & Cubasch, U. MurCSS: A Tool for Standardized Evaluation of Decadal Hindcast Systems. *J. Open Res. Softw.* **2**, 10.5334/jors.bf (2014).

[56] Wilks DS (2016). “The Stippling Shows Statistically Significant Grid Points”: How Research Results are Routinely Overstated and Overinterpreted, and What to Do about It. Bull. Am. Meteorol. Soc..

[57] Maher N, McGregor S, England MH, Gupta AS (2015). Effects of volcanism on tropical variability. Geophys. Res. Lett..

[58] Liu, Y. CESM1.0.5 model output from ‘perfect model’ decadal prediction experiment v1.0. *NCI National Research Data Collection*10.4225/41/5b9fa8780fdfa (2018).

[59] Morice, C. P., Kennedy, J. J., Rayner, N. A. & Jones, P. D. Quantifying uncertainties in global and regional temperature change using an ensemble of observational estimates: The HadCRUT4 data set. *J. Geophys. Res-Atmos.* **117**, 10.1029/2011jd017187 (2012).

