# Author Correction: Enhanced multi-year predictability after El Niño and La Niña events

**DOI:** 10.1038/s41467-023-43407-8

**Published:** 2023-11-27

**Authors:** Yiling Liu, Markus. G. Donat, Matthew. H. England, Lisa. V. Alexander, Annette L. Hirsch, Carlos Delgado-Torres

**Affiliations:** 1 Climate Change Research Centre and ARC Centre of Excellence for Climate Extremes, UNSW, Sydney, NSW 2052 Australia; 2 National Computational Infrastructure (NCI), The Australian National University, Canberra, ACT 2601 Australia; 3 Barcelona Supercomputing Center (BSC), Barcelona, Spain; 4 Institució Catalana de Recerca i Estudis Avançats (ICREA), Barcelona, Spain; 5 Centre for Marine Science and Innovation and Australian Centre for Excellence in Antarctic Science, UNSW, Sydney, NSW 2052 Australia

**Keywords:** Atmospheric dynamics, Projection and prediction

Correction to: *Nature Communications*; 10.1038/s41467-023-42113-9, published online 11 October 2023

The original version of this Article contained an error in ref. 45, which was incorrectly citing the preprint of the article as: Strømmen, K. et al. Seasonal forecasts of the 20th Century. Bullet. Am. Meteorol. Soc. preprint, 10.1175/bams-d-19-0019.1 (2020). The correct form of ref. 45 is: Weisheimer, A. et al. Seasonal Forecasts of the Twentieth Century. *Bull. Am. Meteorol. Soc.* **101**, E1413–E1426 10.1175/bams-d-19-0019.1 (2020). This has been corrected in the PDF and HTML versions of the Article.

