# Supplementary material for: Enhanced multi-year predictability after El Niño and La Niña events
Source: Nat Commun. 2023 Oct 11;14:6387. doi: 10.1038/s41467-023-42113-9 (PMC10567839; doi:10.1038/s41467-023-42113-9)
Supplement: Supplementary file 1 — Supplementary Information [file 41467_2023_42113_MOESM1_ESM.pdf]

Supplementary Information

Enhanced multi-year predictability after El Niño and La Niña events

Yiling Liu[1,2*], Markus. G. Donat[3,4], Matthew. H. England[5], Lisa. V. Alexander[1], Annette L. Hirsch[1], Carlos Delgado-Torres[3]

[1] Climate Change Research Centre and ARC Centre of Excellence for Climate Extremes, UNSW Sydney, NSW 2052, Australia

[2] National Computational Infrastructure (NCI), The Australian National University, ACT 2601, Australia

[3] Barcelona Supercomputing Center (BSC), Barcelona, Spain

[4] Institució Catalana de Recerca i Estudis Avançats (ICREA), Barcelona, Spain

[5] Centre for Marine Science and Innovation and Australian Centre for Excellence in Antarctic Science, UNSW Sydney, NSW 2052, Australia

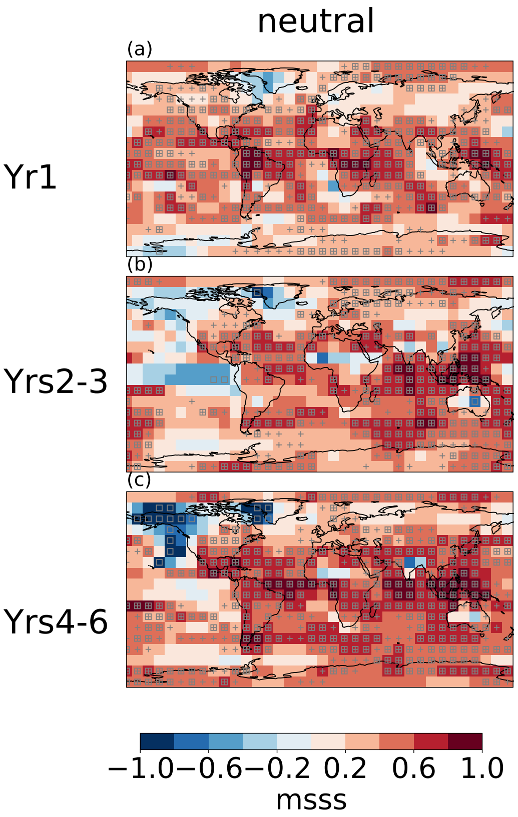

Figure S1 The mean squared skill score (MSSS) for the near-surface temperature in the perfect-model predictions for decadal simulations initialised in neutral conditions (a for Year 1; b for Years 2-3; c for Years 4-6), complementing the results shown in Figure 1.

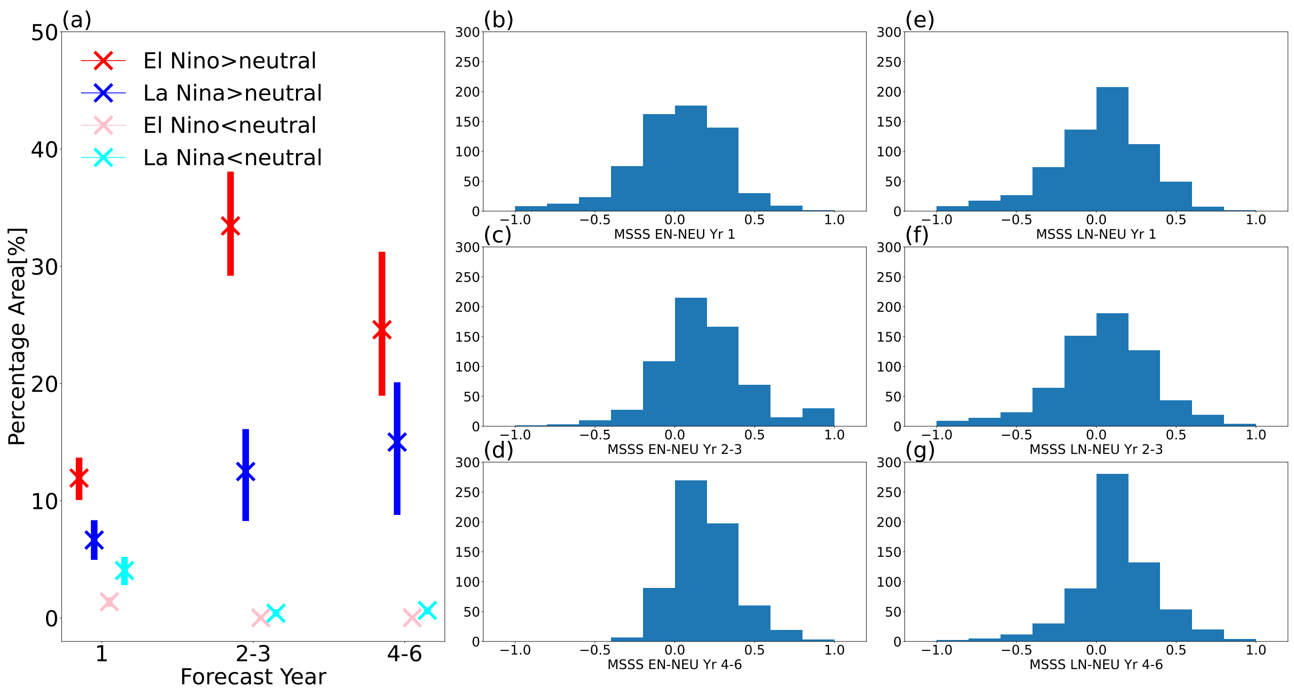

Figure S2 (Left, a) Percentage of global area in the perfect-model predictions where the mean squared skill score (MSSS) differences between prediction started in El Niño or La Niña conditions and neutral conditions are locally outside the 5-95% confidence interval based on 1,000 bootstrap realisations. Red symbols (El Niño>neutral) represent the area in which the skill of predictions started in El Niño conditions is significantly larger than the skill of predictions started in neutral conditions, blue symbols (La Niña>neutral) the area where predictions started in La Niña conditions are more skilful than predictions started in neutral conditions; the light red/light blue symbol indicate accordingly the area where predictions started in El Niño/La Niña conditions are less skilful than predictions started in neutral conditions. The vertical bars represent the 5th-95th percentile range based on 1,000 times drawing random samples of 100 out of the 1,000 bootstrap realisations. (Right, b to g) Histograms of the MSSS difference values (across all grid cells) between predictions initialised in El Niño (EN)/La Niña (LN) and neutral (NEU) conditions for El Niño minus Neutral in year 1 (b); El Niño minus Neutral in years 2-3 (c); El Niño minus Neutral in years 4-6 (d); La Niña minus Neutral in year 1 (e); La Niña minus Neutral in years 2-3 (f); La Niña minus Neutral in years 4-6 (g).

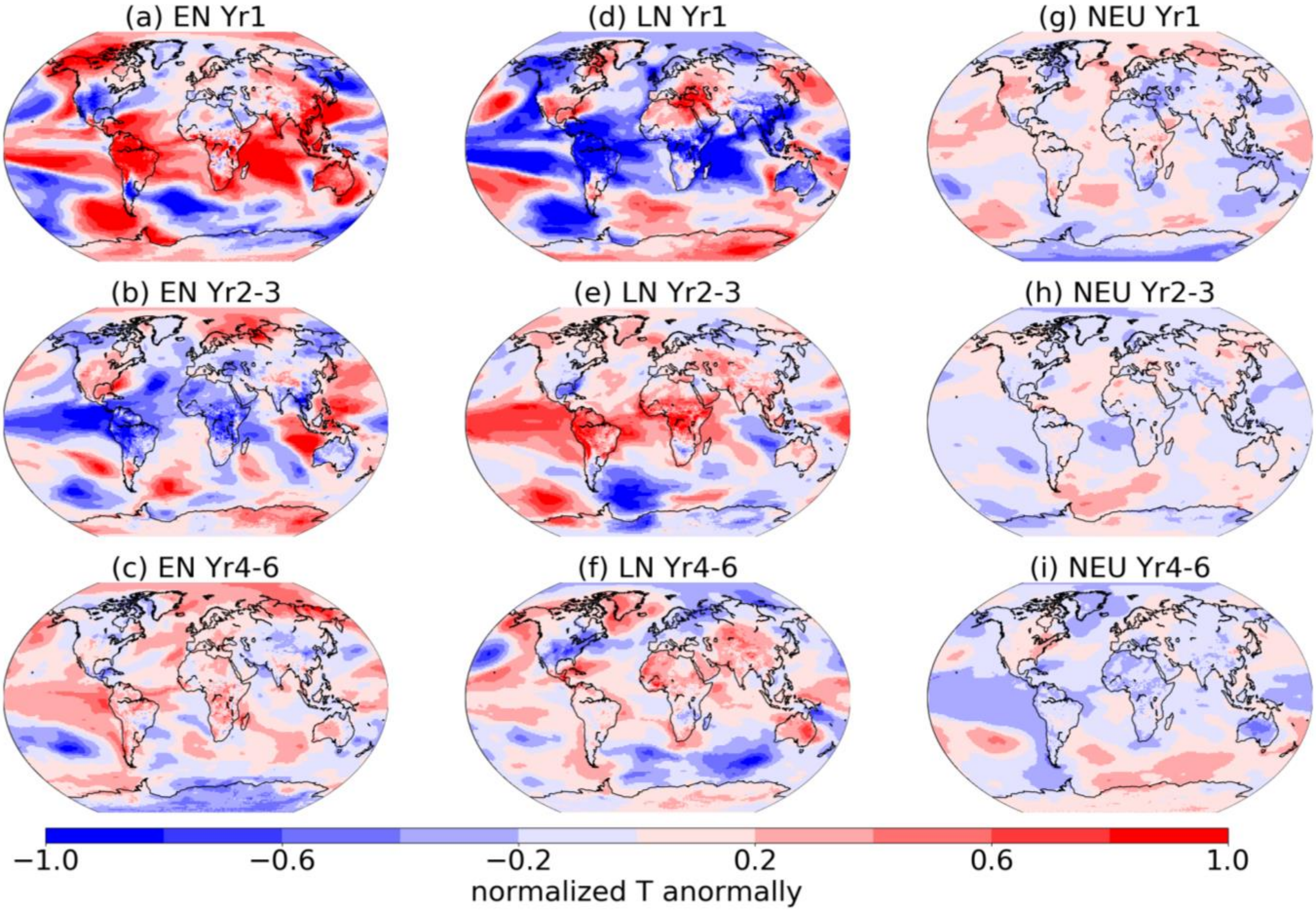

Figure S3 Same as Fig. 3 but for the normalized temperature from the perfect-model reference run. No pattern correlation values are shown here as these patterns are used for reference when calculating the correlations in Fig. 3 and S3.

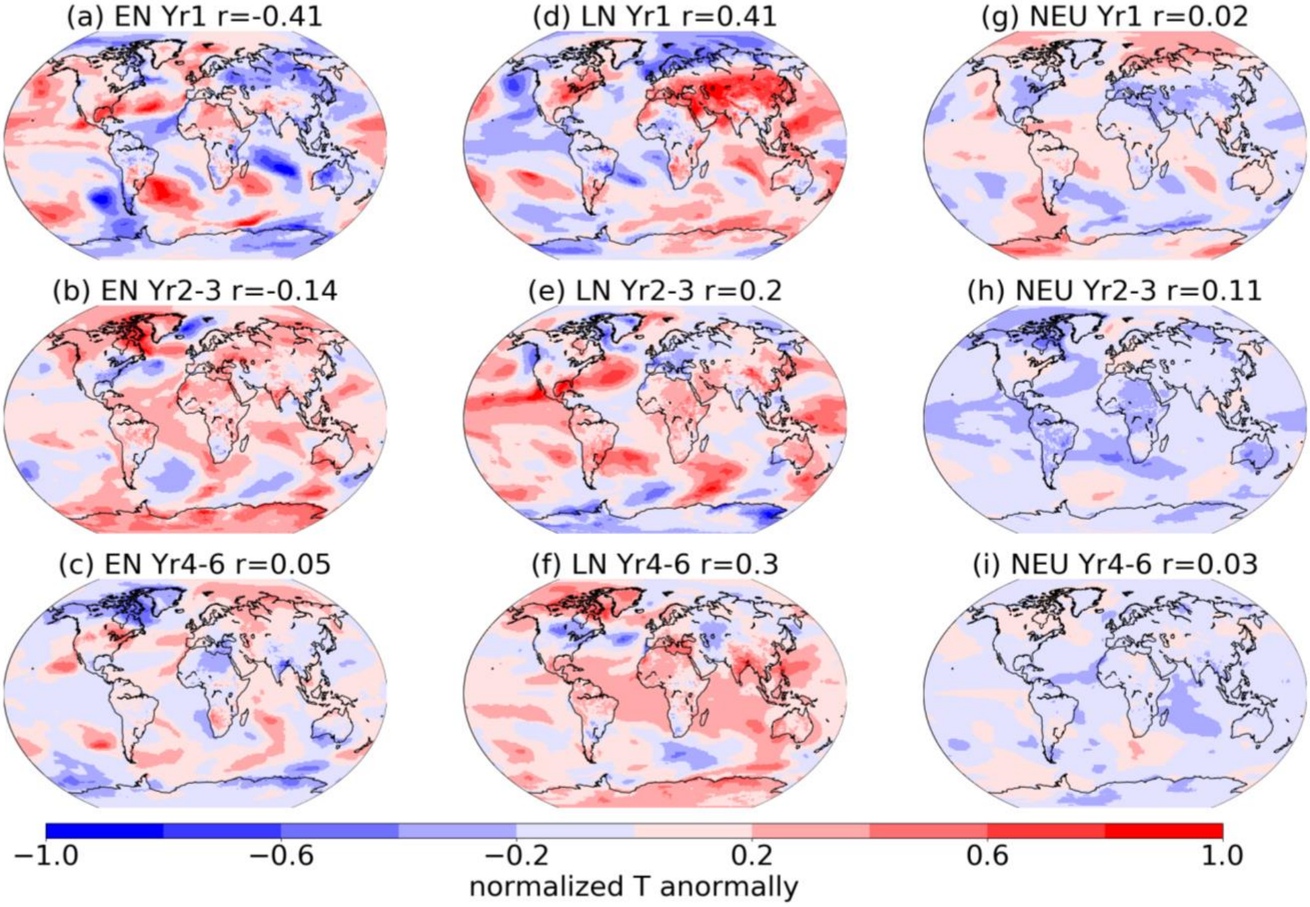

Figure S4 Same as Fig. 3 but for the normalized temperature from the uninitialized perfect-model predictions. The r numbers in the title show the pattern correlation between uninitialized historical simulations ensemble mean and the reference run.

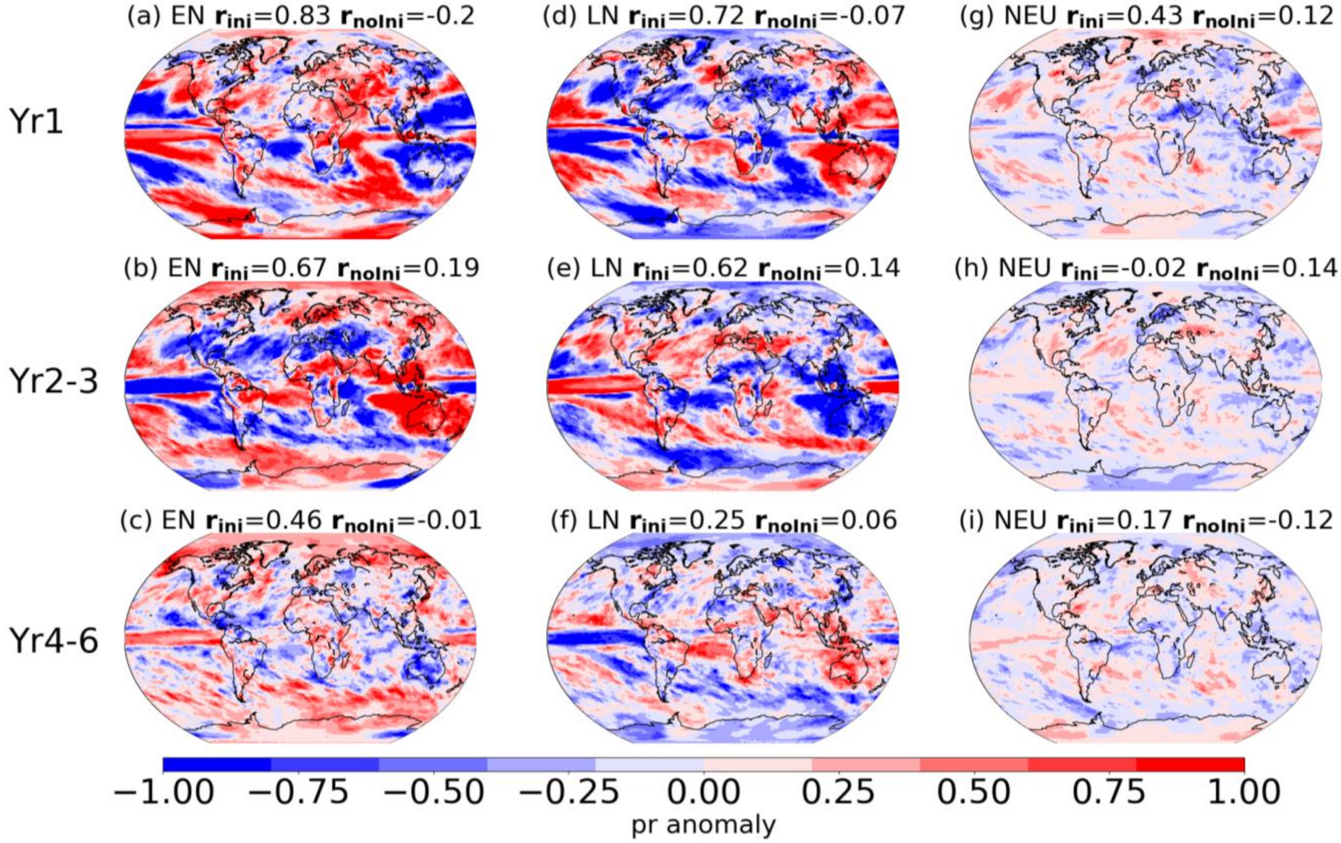

Figure S5 Same as Fig. 3 but for precipitation.

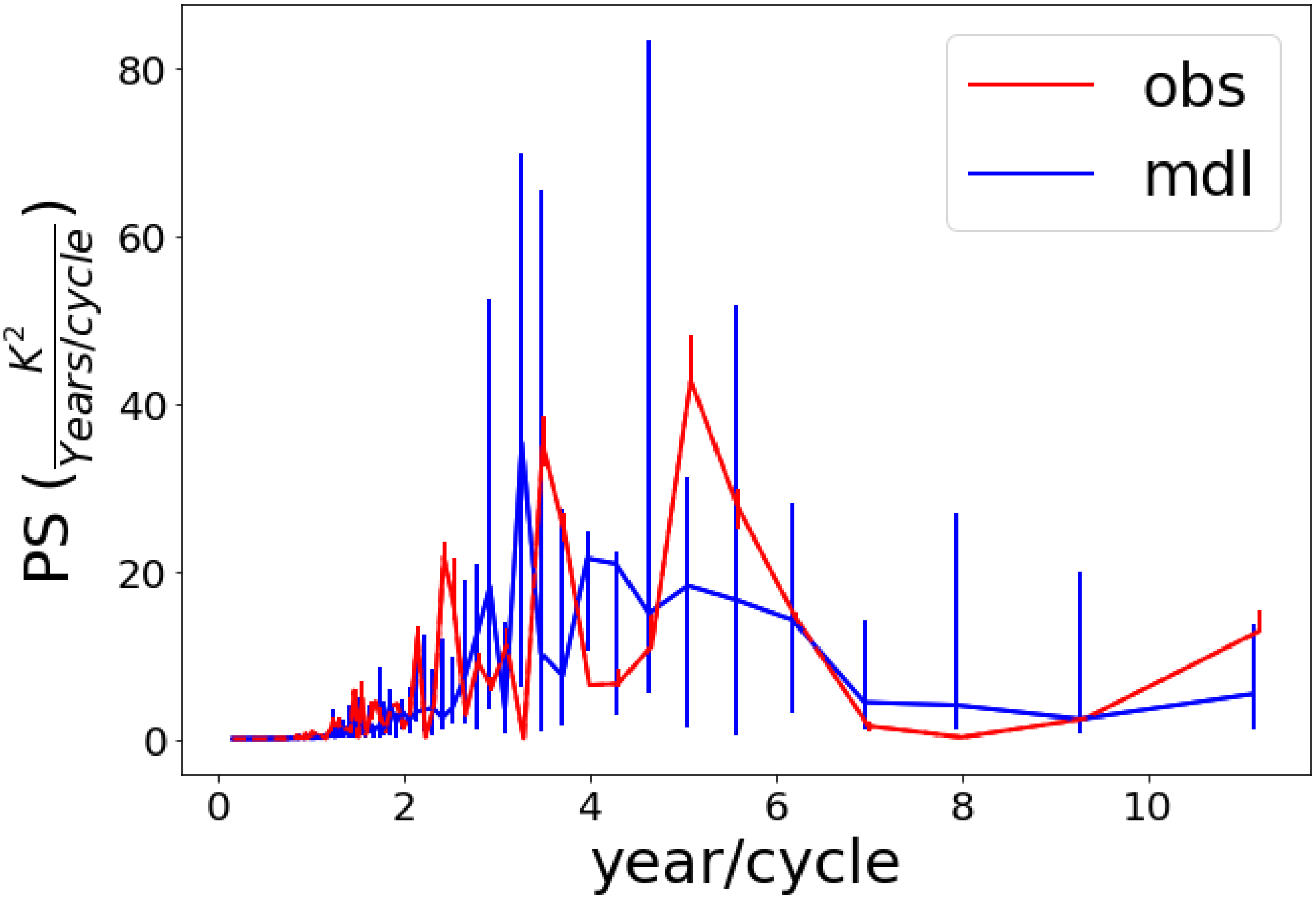

Figure S6 Power spectrum (PS) of the detrended Nino34 index in the Community Climate System Model Version 4 (CCSM4) model (mdl; blue curve) and the observation (obs; red curve) during 1950-2005. The blue curve shows the median of the power spectrum from six CCSM4 historical simulations within the Coupled Model Intercomparison Project Phase 5 (CMIP5) archive in addition to one extra perfect-model reference simulation conducted to initialize the perfect-model predictions[1]. The red curve shows the median of the power spectrum from three observational datasets -- the Hadley Centre Global Sea Ice and Sea Surface Temperature (HadISST)[2]; the Goddard Institute for Space Studies surface temperature analysis (GISTEMP)[3] and National Oceanic and Atmospheric Administration's Merged Land - Ocean Surface Temperature Analysis (MLOST)[4,5]. The vertical bars denote the spread across all ensemble members (seven members for the model and three members for the observation).

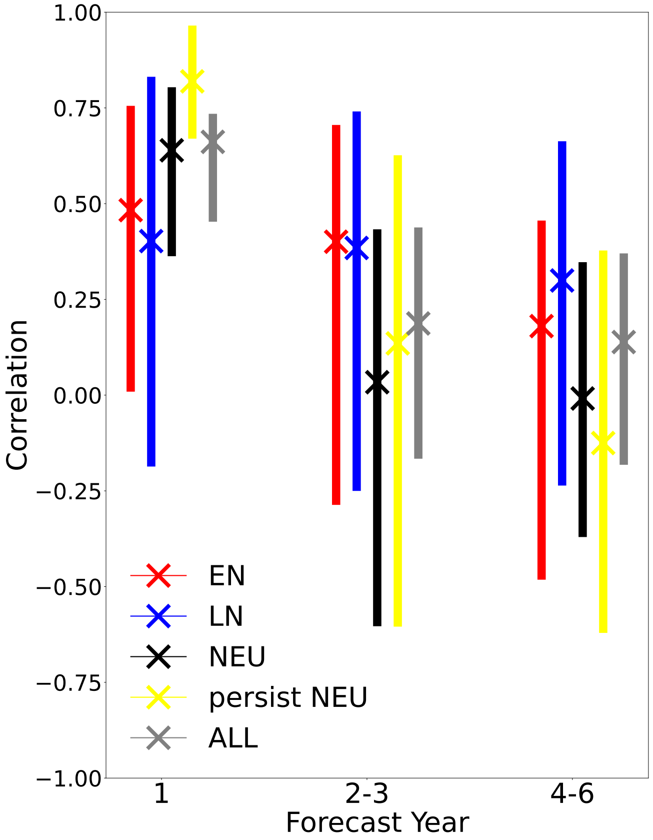

Figure S7 Predictability of El Niño-Southern Oscillation (ENSO) itself. The correlation of the Niño3.4 index for predictions started at the different initial states for the different forecasting periods (years 1, 2-3 and 4-6). Red symbols represent predictions start from El Niño conditions (EN); blue symbols are for La Niña starting conditions (LN); black symbols are for neutral starting years (NEU); yellow symbols for persistent neutral starting years (persistent NEU); and grey for all starting years (ALL). The vertical bars represent the 5 th-95th percentile ranges based on 1,000 bootstrap realisations.

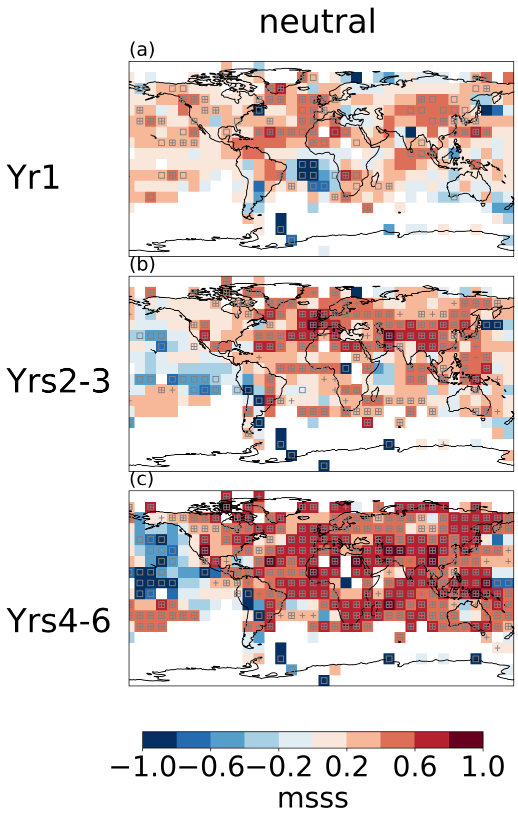

Figure S8 The mean squared skill score (MSSS) for the near-surface temperature in the Community Earth System Model decadal prediction large ensemble (CESM-DPLE) decadal hindcasts initialised in neutral conditions (a for year 1; b for years 2-3; c for years 4-6), complementing the results shown in Figure 4.

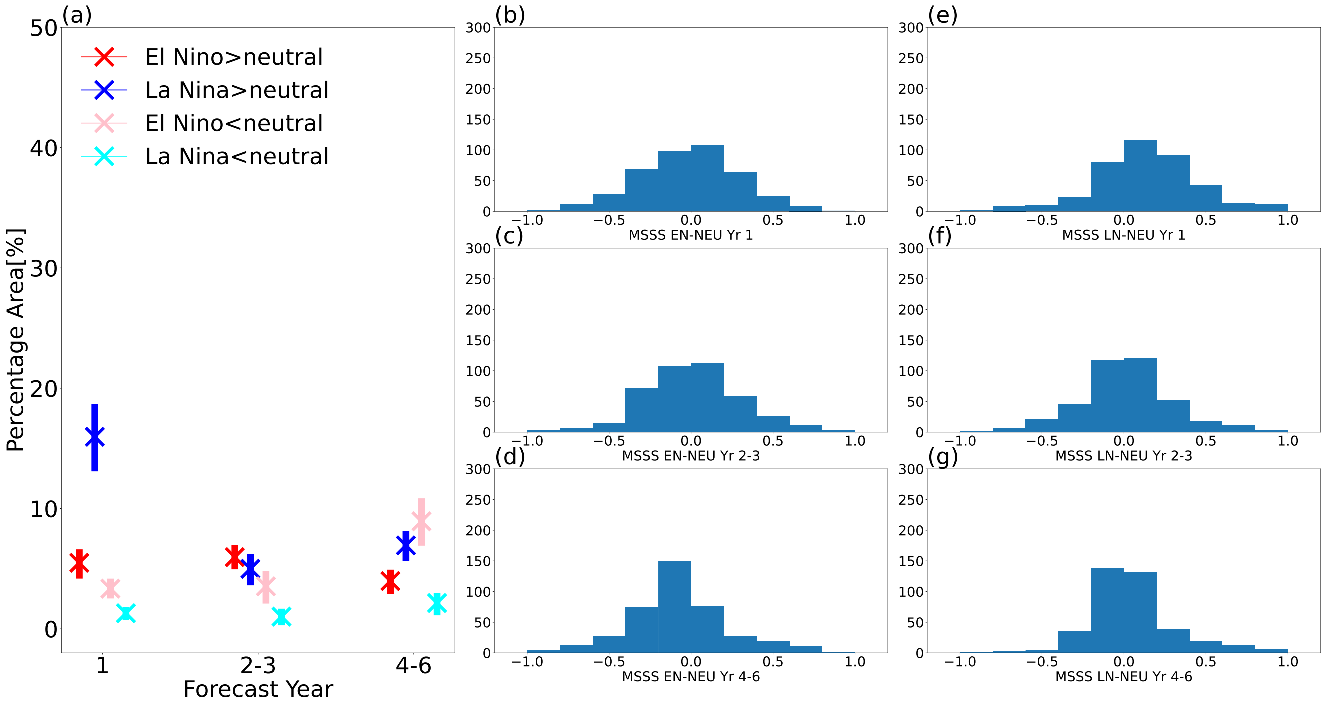

Figure S9 Same as Figure S2 but for the real-world hindcasts with the Community Earth System Model decadal prediction large ensemble (CESM-DPLE).

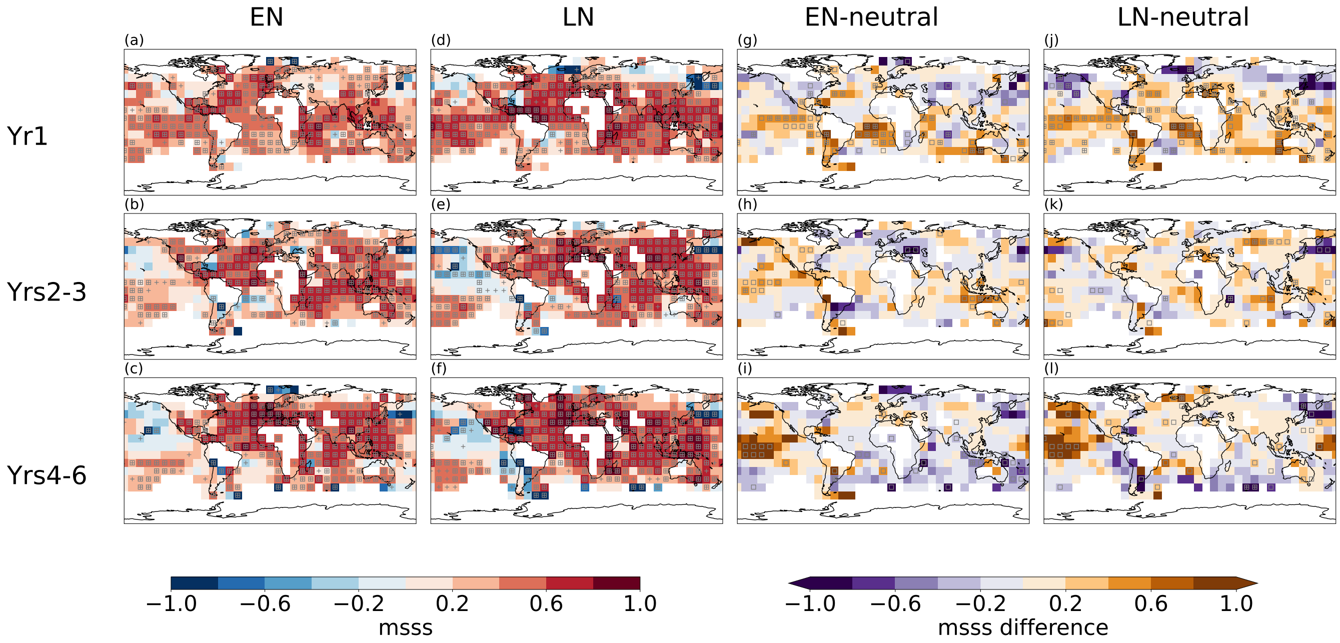

Figure S10 Same as Figure 4 but for the multi-model ensemble of decadal hindcasts provided within the Coupled Model Intercomparison Project Phase 6 Decadal Climate Prediction Project. (CMIP6-DCPP). See Supplementary Table S4 for a list of the models included.

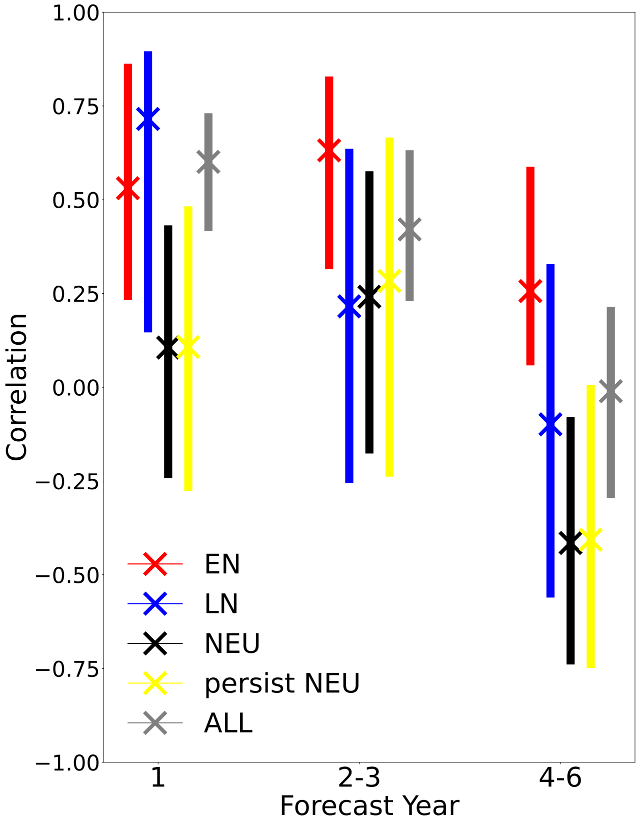

Figure S11 Same as Figure S7 but for the real-world hindcasts with the Community Earth System Model decadal prediction large ensemble (CESM-DPLE).

Table S1 Classification of starting years for the reference simulation from which the perfect-model predictions are started, and classification of observed El Niño-Southern Oscillation (ENSO) years to divide the real-world predictions accordingly.

(a) perfect- model predictions

El Niño	1963,1967,1973,1977,1983,1989,1995,1999,2000,2001,2003,2006
La Niña	1961,1965,1966,1969,1971,1974,1978,1981,1985,1986,1996,1997,2005
neutral	1962,1964,1968,1970,1972,1975,1976,1979,1980,1982,1984,1987,1988,1990,1991,1992,1993,1994, 1998,2002,2004
persistent neutral	1964,1975,1980,1982,1984,1987,1988,1990,1991,1992,1994,2002

(b) real-world predictions

El Niño	1957,1963,1965,1968,1969,1972,1976,1977,1979,1982,1986,1987,1991,1994,1997,2002
La Niña	1954,1955,1964,1970,1973,1975,1983,1984,1988,1995,1998,1999,2000,2005
neutral	1956,1958,1959,1960,1961,1962,1966,1967,1971,1974,1978,1980,1981,1985,1989,1990,1992,1993,1996,2001,2003,2004,2006
persistent neutral	1958,1959,1960,1961,1962,1966,1967,1974,1978,1980,1981,1989,1990,1992,1993,1996,2001,2003

Table S2 El Niño-Southern Oscillation (ENSO) transition probabilities for perfect-model predictions. The transition probabilities are determined from the five initialized prediction members (initialized in model years 1961-2005), conditional on the ENSO phase (El Niño, La Niña and neutral) at initialization of the predictions, for forecast years 1, forecast years 2-3, and forecast years 4-6.

	forecast year 1			forecast years 2 or 3			forecast years 4, 5 or 6		
Initial ENSO phase	To El Niño	To La Niña	To neutral	To El Niño	To La Niña	To neutral	To El Niño	To La Niña	To neutral
El Niño	17%	48%	35%	19%	32%	49%	28%	25%	47%
La Niña	26%	11%	63%	37%	22%	41%	22%	33%	45%
Neutral	24%	31%	45%	29%	27%	44%	29%	28%	43%

Table S3 El Niño-Southern Oscillation (ENSO) transition probabilities in observations (HadISST 1.1[2]). Transitions are counted 1, 2 or 3, and 4, 5 or 6 years after years classified as El Niño, La Niña and neutral year. The Niño3.4 index was derived from HadISST 1.1 data between 1900 to 2015, where El Niño events are defined when Niño3.4 temperatures are in the upper quartile of the distribution and La Niña events are defined when Niño3.4 temperatures are in the lower quartile of the distribution (quartile thresholds are calculated from data during 1960-2015, as for the model simulations).

	after 1 year			after 2 or 3 years			after 4, 5 or 6 years		
Initial ENSO phase	To El Niño	To La Niña	To neutral	To El Niño	To La Niña	To neutral	To El Niño	To La Niña	To neutral
El Niño	21%	28%	51%	19%	21%	60%	22%	23%	55%
La Niña	22%	26%	52%	33%	19%	48%	20%	21%	59%
Neutral	25%	16%	59%	21%	24%	55%	23%	24%	53%

	after 5, 6 or 7 years		
Initial ENSO phase	To El Niño	To La Niña	To neutral
El Niño	25%	22%	53%
La Niña	17%	24%	59%
Neutral	22%	23%	55%

Table S4 Overview of models included in the the Coupled Model Intercomparison Project Phase 6 Decadal Climate Prediction Project. (CMIP6-DCPP) multi-model analysis of real-world decadal hindcasts, and some key characteristics of the prediction systems.

**Forecast system and reference**	**DCPP members**	**Spatial resolution**	**Month initialization**
BCC-CSM2-MR[6]	8	1.125°x1.125°	January
CanESM5[7]	20	2.8°x2.8°	January
CMCC-CM2-SR5[8]	10	0.9°x1.25°	November
EC-Earth3-i1[9,10]	10	0.7°x0.7°	November
EC-Earth3-i2[10]	5	0.7°x0.7°	November
EC-Earth3-i4[10]	10	0.7°x0.7°	November
HadGEM3-GC3.1-MM[11]	10	0.55°x0.83°	November
IPSL-CM6A-LR[12]	10	1.25°x2.5°	January
MIROC6[13]	10	1.4°x1.4°	November
MPI-ESM1.2-HR[14]	10	0.9°x0.9°	November
MRI-ESM2-0[15]	10	1.125°x1.125°	November

NorCPM1-il[16]	10	1.9°x2.5°	October
NorCPM1-i2[16]	10	1.9°x2.5°	October

Supplementary References:

1. Liu, Y. *et al.* A Framework to Determine the Limits of Achievable Skill for Interannual to Decadal Climate Predictions. *Journal of Geophysical Research: Atmospheres* **124**, 2882-2896, doi:10.1029/2018jd029541 (2019).
2. Rayner, N. A. Global analyses of sea surface temperature, sea ice, and night marine air temperature since the late nineteenth century. *Journal of Geophysical Research* **108**, doi:10.1029/2002jd002670 (2003).
3. Hansen, J., Ruedy, R., Sato, M. & Lo, K. Global Surface Temperature Change. *Reviews of Geophysics* **48**, doi:10.1029/2010rg000345 (2010).
4. Smith, T. M., Reynolds, R. W., Peterson, T. C. & Lawrimore, J. Improvements to NOAA's Historical Merged Land–Ocean Surface Temperature Analysis (1880–2006). *Journal of Climate* **21**, 2283-2296, doi:10.1175/2007jcli2100.1 (2008).
5. Vose, R. S. *et al.* NOAA's Merged Land–Ocean Surface Temperature Analysis. *Bulletin of the American Meteorological Society* **93**, 1677-1685, doi:10.1175/bams-d-11-00241.1 (2012).
6. Wu, T. *et al.* The Beijing Climate Center Climate System Model (BCC-CSM): the main progress from CMIP5 to CMIP6. *Geosci Model Dev* **12**, 1573-1600, doi:10.5194/gmd-12-1573-2019 (2019).
7. Swart, N. C. *et al.* The Canadian Earth System Model version 5 (CanESM5.0.3). *Geosci Model Dev* **12**, 4823-4873, doi:10.5194/gmd-12-4823-2019 (2019).
8. Nicolì, D. *et al.* The Euro-Mediterranean Center on Climate Change (CMCC) decadal prediction system. *Geosci Model Dev* **16**, 179-197, doi:10.5194/gmd-16-179-2023 (2023).
9. Bilbao, R. *et al.* Assessment of a full-field initialized decadal climate prediction system with the CMIP6 version of EC-Earth. *Earth Syst Dynam* **12**, 173-196, doi:10.5194/esd-12-173-2021 (2021).
10. Döscher, R. *et al.* The EC-Earth3 Earth system model for the Coupled Model Intercomparison Project 6. *Geosci Model Dev* **15**, 2973-3020, doi:10.5194/gmd-15-2973-2022 (2022).
11. Sellar, A. A. *et al.* Implementation of U.K. Earth System Models for CMIP6. *J Adv Model Earth Sy* **12**, doi:10.1029/2019ms001946 (2020).
12. Boucher, O. *et al.* Presentation and Evaluation of the IPSL‐CM6A‐LR Climate Model. *J Adv Model Earth Sy* **12**, doi:10.1029/2019ms002010 (2020).
13. Tatebe, H. *et al.* Description and basic evaluation of simulated mean state, internal variability, and climate sensitivity in MIROC6. *Geosci Model Dev* **12**, 2727-2765, doi:10.5194/gmd-12-2727-2019 (2019).
14. Müller, W. A. *et al.* A Higher‐resolution Version of the Max Planck Institute Earth System Model (MPI‐ESM1.2‐HR). *J Adv Model Earth Sy* **10**, 1383-1413, doi:10.1029/2017ms001217 (2018).
15. Yukimoto, S. *et al.* The Meteorological Research Institute Earth System Model Version 2.0, MRI-ESM2.0: Description and Basic Evaluation of the Physical Component. *Journal of the Meteorological Society of Japan. Ser. II* **97**, 931-965, doi:10.2151/jmsj.2019-051 (2019).
16. Bethke, I. *et al.* NorCPM1 and its contribution to CMIP6 DCPP. *Geosci Model Dev* **14**, 7073-7116, doi:10.5194/gmd-14-7073-2021 (2021).
